# Immune Senescence, Immunosenescence and Aging

**DOI:** 10.3389/fragi.2022.900028

**Published:** 2022-05-30

**Authors:** Kyoo-A Lee, Rafael R. Flores, In Hwa Jang, Ashley Saathoff, Paul D. Robbins

**Affiliations:** Institute on the Biology of Aging and Metabolism, Department of Biochemistry, Molecular Biology and Biophysics, University of Minnesota, Minneapolis, MN, United States

**Keywords:** senescence, immunosenescence, senolytic, aging, immunity

## Abstract

With aging, there is increased dysfunction of both innate and adaptive immune responses, which contributes to impaired immune responses to pathogens and greater mortality and morbidity. This age-related immune dysfunction is defined in general as immunosenescence and includes an increase in the number of memory T cells, loss of ability to respond to antigen and a lingering level of low-grade inflammation. However, certain features of immunosenescence are similar to cellular senescence, which is defined as the irreversible loss of proliferation in response to damage and stress. Importantly, senescence cells can develop an inflammatory senescence-associated secretory phenotype (SASP), that also drives non-autonomous cellular senescence and immune dysfunction. Interestingly, viral infection can increase the extent of immune senescence both directly and indirectly, leading to increased immune dysfunction and inflammation, especially in the elderly. This review focuses on age-related immune dysfunction, cellular senescence and the impaired immune response to pathogens.

## Introduction

### Immunosenescence

Aging causes the gradual decline of immune system function, which results in an increase in the incidence of diseases such as cancer and infections. In general, this age-related immune dysfunction is defined as immunosenescence, characterized by changes in various aspects of different immune components such as thymic involution and loss of diversity of adaptive immunity. Features of immunosenescence include an increase in the number of memory T cells, loss of ability to respond to antigen and a lingering level of low-grade inflammation termed “inflamm-aging.” Also, there are phenotypic changes in multiple immune cell types that serve to define immunosenescence. Importantly, latent and chronic viral infection [e.g., human cytomegalovirus (HCMV), Epstein-Barr virus (EBV)] also affect the immune system and contribute to immunosenescence with age.

### Cellular Senescence

With age, there also is an increase in the number of senescent cells (SnCs). Cellular senescence, a cell fate, is found to increase in most tissues including lymphoid tissues in multiple cell types (Yousefzadeh et al., 2020; Yousefzadeh et al., 2021). Cellular senescence is defined as the irreversible exit from the cell cycle in response to various types of stress such as uncontrolled DNA replication and genotoxic, oxidative and inflammatory stress. SnCs are characterized by evidence of telomere associate foci (TAFs), senescence-associated distension of satellites (SADS), senescence-associated heterochromatin foci (SAHF), senescence-associated ß-galactosidase (SA-ß-gal) and upregulation of at least 1 cell cycle dependent kinase inhibitor (e.g., p16^INK4a^, p21^Cip1^) (Robbins et al., 2021). In addition, many SnCs develop a pro-inflammatory senescence-associate secretory phenotype (SASP) that includes secretion of various cytokines and chemokines that recruit or modulate immune cell function (Rodier et al., 2009). Secretion of metalloproteinases, miRNAs, ROS, metabolites and even extracellular vesicles also can be part of the SASP. SnCs accumulate in aged tissues where they are responsible for local and systemic inflammation *via* the SASP, which leads to stem cell dysfunction and age-associated tissue degeneration (Rodier et al., 2009; Coppe et al., 2010).

Senescence is thought to act as a robust tumor suppressor mechanism, with the SASP acting as a chemoattractant to stimulate immune cell-mediated clearance of SnCs (Coppe et al., 2010). However, with advancing age and in many chronic diseases such as obesity and metabolic syndromes, SnCs accumulate in most tissues, presumably due to inefficient SnC removal by the immune system and increased resistance to cell death. This accumulation drives chronic sterile inflammation *via* SASP, which in turn drives loss of resilience and predisposition to many diseases. In this context, senescence can even drive cancer (Eggert et al., 2016). SnCs also can interfere with the immune system and the ability of immune cells to remove them (Prata et al., 2018). For example, certain SASP factors alter immune cell migration and matrix metalloproteinases cleave immune regulators on the cell surface (Kayagaki et al., 1995; Prata et al., 2018).

SnCs also play a causal role in aging and age-related diseases in pre-clinical models (Baker et al., 2011; Baker et al., 2016). For example, transplanting SnCs into young mice causes an accelerated aging-like state, while genetic or pharmacologic selective killing of SnCs attenuates disease, improves physical function, and delays all-cause mortality in older mice (Xu et al., 2018; Yousefzadeh et al., 2018). Interestingly, although many of the SASP factors are expressed by SnCs at steady state level, the level of SASP expression substantially increases in response to innate immune cues, such as exposure to pathogen- and damage-associated molecular patterns (PAMPs and DAMPs) (Camell et al., 2021; Tripathi et al., 2021). While at first glance this may appear beneficial for reactivity to pathogens, excessive generation of SASP factors can contribute to hyperinflammation or the “cytokine storm” associated with pathogenic responses to immune stimuli (Camell et al., 2021). Importantly, viral infection itself is able to induce senescence, termed virus-induced senescence (VIS). VIS is universal stress response in host cells infected by a large variety of different virus species, including single- and double-stranded DNA and RNA viruses (Chuprin et al., 2013; Li et al., 2013; Martínez et al., 2016; Kohli et al., 2021; Lee et al., 2021). The programmed VIS response is morphologically and transcriptionally indistinguishable from other types of senescence including oncogene-induced senescence (OIS) (Serrano et al., 1997; Braig et al., 2005).

**FIGURE 1:**
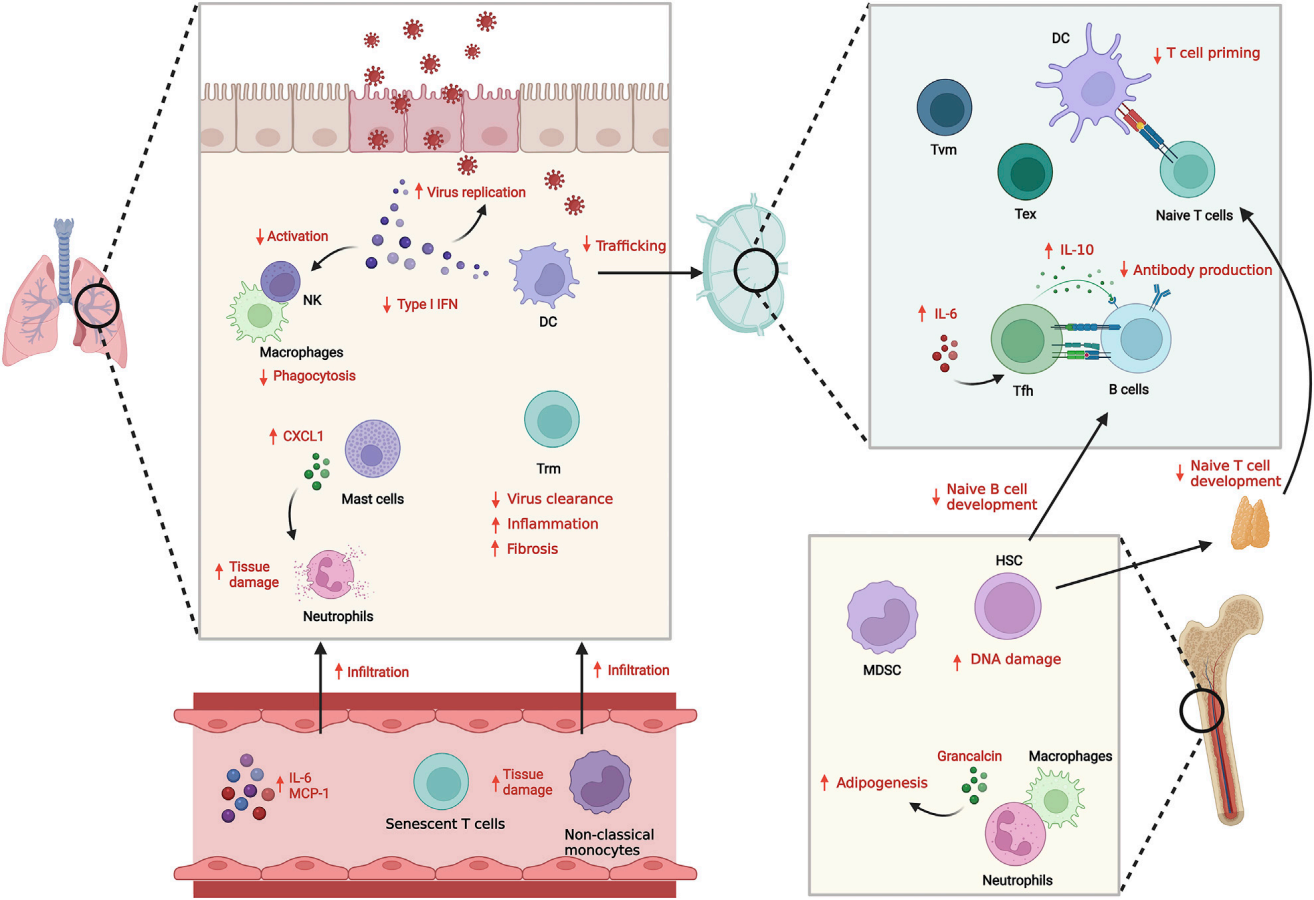
Innate and adaptive immune system dysfunction with aging and infection. With aging, both innate and adaptive immunity show dysregulated responses against infection, resulting in suboptimal anti-pathogenic responses and increased inflammation. The reduced Type I IFN secretion and functional defects in innate immune cells including senescence results in inefficient clearance of pathogens. Defects in DC trafficking and T cell priming also cause impaired adaptive immune response including antibody generation and cellular T cell responses. Instead, infiltration of inflammatory senescent immune cells is increased, which induces tissue damage.

Although cellular senescence was originally defined in fibroblasts and other cell types in solid organs, there is increasing evidence that immune cells also can show signs of senescence. However, senescence in immune cells has not fully characterized or defined due to the diversity of function and phenotypes of each immune cell type as they differentiate. In this review, we discuss senescence, immunosenescence and immune dysfunction with age and disease, in particular, bacterial and viral infection (Figure 1).

## Innate and Adaptive Immune Dysfunction With Age During Infection

### Innate Immunity

Innate immunity is mediated by phagocytic cells, including macrophages. dendritic cells (DC), and granulocytes as well as innate or innate-like lymphocytes such as natural killer (NK) cells, γδ T cells. These cell types provide an immediate, non-antigen specific response to viral, bacterial and fungal pathogens. In addition, the type I IFN (IFNα and IFNβ) response, mediated by recognition of PAMPs through pattern recognition receptors (PRR) such as RIG-1/MDA-5, is a first line of defense against pathogens by inhibiting viral replication and spread at the early stage of infection. The extent of type I IFN response determines the susceptibility to viral infections. For example, COVID-19 patients with higher levels of type I IFN neutralizing auto-antibodies are at a higher risk for life-threatening pneumonia (Bastard et al., 2020).

There is a both a reduction and delay in type I IFN production with age due to impaired signaling of the receptors and reduced number of circulating plasmacytoid dendritic cells (pDCs), a source of type I IFNs (Molony et al., 2017; Feng et al., 2021). Given that type I IFNs also recruit and activate immune cells like macrophages and NK cells (Acharya et al., 2020), this reduction in type I IFNs affects the aged innate immune response at multiple levels.

Dendritic cells (DCs) process antigens and initiate adaptive immunity in draining lymph nodes (DLNs). In murine respiratory virus infection models, lung DCs in aged mice show defect in migration to DLNs leading to lower T cell responses (Zhao et al., 2011). In severe pulmonary virus infection such as influenza A virus (IAV) and SARS-CoV-2, excessive neutrophil infiltration is observed in lung, which induces tissue damage (Feng et al., 2021). This could be caused by reduced type I IFN that suppresses neutrophil recruitment. In addition, aged alveolar macrophages show decreased ability to phagocytose neutrophils, resulting in increased retention of neutrophils and tissue damage during influenza infection (Wong et al., 2017).

There is also an age-dependent increase in the number of myeloid-derived suppressor cells (MDSCs) that are able to inhibit T cell function and thus contribute to the pathogenesis of various diseases including cancer and infectious diseases. In mouse aging, there is an increase in the percent of Gr-1^+^ CD11b^+^ MDSCs in bone marrow with an enhanced NF-κB activation, likely resulting in the cells being more inflammatory (Flores et al., 2017). Given that a reduction in NF-κB activity reduces the percent of MDSCs, it is possible the activation of NF-κB with aging and senescence-like cell status drives this increase in the percentage of MDSCs.

### Adaptive Immunity

The adaptive immune response is mediated predominantly by T cells and B cells, which play critical roles in the clearance of pathogens as well as in the response to vaccination. The adaptive immune response wanes with age, even in rodent models not exposed to pathogens. The reduced B cell response with aging results in reduced quality and quantity of the antibody response to infections. Aged B cells produce less antibodies and with skewing towards antibodies with lower affinity, leading to suboptimal protection against new infections and reduced efficacy to vaccine (Borgoni et al., 2021). There is also a reduction in the number of naïve B cells and an increase in memory B cells with age. For adequate antibody generation, help from follicular helper T (Tfh) cells is required. With age, the systemic increase of IL-10 from Tfh cells limits the response to vaccination and antibody production. IL-6 and IL-21 are required for maintenance of IL-10 producing Tfh cells, suggesting that SASP factors such as IL-6 produced by senescent cells that accumulate with age might be involved in modulation of Tfh cells (Almanan et al., 2020).

CD8^+^ cytotoxic T cells are important for the facilitating clearance of pathogens by the selective killing of infected cells. However, there is a reduction in the number and repertoire of CD8^+^ cells with aging. In addition, there is a narrowing of the repertoire of influenza specific conventional αβ CD8^+^ T cells with large random clonal expansion and reduced use of the public clonotypes (Gil et al., 2015; Nguyen et al., 2018). In addition, elderly COVID-19 patients (>80 years of age) show diminished T cells with cytotoxic profiles (Westmeier et al., 2020).

Tissue resident memory T (Trm) cells, a type of memory cell that resides in tissues permanently, are important for control infections and likely cancer. Even in unexposed subjects, SARS-CoV-2 reactive CD8 Trm cells have been identified in tissues (Niessl et al., 2021) that are poised to respond to pathogens. However, with aging, these Trms are less effective in clearing infection and, even following clearance of pathogen, drive persistent inflammation and fibrosis (Goplen et al., 2020).

## Cellular Senescence in Immune Cells

As discussed below, there is evidence of senescence in different types of immune cells. Interestingly, the aged immune system not only has altered development and reduced function, but also can induce senescence of peripheral tissues and tissue damage through a non-autonomous mechanism. For example, a recent study demonstrated that deletion of Ercc1, part of the endonuclease Ercc1-XPF important in multiple types of DNA repair, specifically in hematopoietic cells resulted in the accumulation of spontaneous DNA damage in immune cells and increased expression of markers of senescence and SASP in a variety of immune cell types including CD4^+^ and CD8^+^ T, B and NK cells and in monocyte/macrophages, similar to that observed in two-year-old naturally aged mice (Yousefzadeh et al., 2021). These mice with aged, senescent immune cells showed several features of aging such as shortened lifespan and multi-organ tissue degeneration. The mice also had an increase in the level of senescence in non-immune organs including liver, kidney, intervertebral discs and muscle. In addition, the adoptive transfer of splenocytes from aged WT mice into young mice also drove the induction of senescence and SASP makers in different tissues as well as shortened lifespan (Yousefzadeh et al., 2021). These results suggest that DNA damage can increase the percent of senescent immune cells with age, which then can drive secondary senescence through secretion of SASP factors. However, the specific subset(s) of immune cells that drive systemic senescence in lymphoid and non-lymphoid tissues and aging still need to be determined. In contrast, the adoptive transfer of young immune cells into a mouse model of accelerated senescence and aging resulted in a reduction in the senescent cell burden in multiple tissues, suggesting that young immune cells can clear SnCs that arise with aging and disease.

### Senescence in T Cells

With human aging, there is accumulation of CD28^−^ T cells, especially in the CD8^+^ T cell population (Weng et al., 2009). Highly differentiated CD27^−^ CD28^−^ CD8^+^ T cells displays senescent-like features such as reduced proliferation rate, shorter telomere and increased level of p38 and γH2AX (Henson et al., 2014). CD45RA^+^CD27^−^CD4^+^ T cells were suggested to be senescent-like T cells with constitutive p38 expression and reduced telomerase activity and proliferation. These senescent-like T cells express NK cell receptors with reduced activating capacity through T cell receptors (TCR). Since these cells express high levels of cytotoxic molecules, they might be responsible for the massive tissue damage *via* NK cell receptors (NKR) rather than specific killing through TCR (Suarez-Álvarez et al., 2017; Pereira et al., 2020).

Recently, an effort to find the “true” senescent immune cells upon human aging with SA-β-gal staining identified aged circulating T cells, in particular, CD8^+^ T cells, as having a senescence gene expression signature as well as reduced proliferative capacity. However, the high SA-β-gal CD8^+^ T cells show a unique T-cell fate, distinct from the cells defined previously as senescent T cells (Martínez-Zamudio et al., 2021). Also, the p16^INK4a^ expression in CD8^+^ T cells was shown to correlate with biological age and disease (Pustavoitau et al., 2016).

With murine aging, there are limited numbers of studies regarding senescent T cells with age. CD8^+^ virtual memory (Tvm) cells that accumulate with age exhibit senescence profiles and reduced proliferation (Quinn et al., 2018). In particular, aged Tvm cells express high Cdkn1a and γ-H2AX with a defect in proliferation in response to TCR stimulation. It has been proposed that CD4^+^ senescent-like T cell can be characterized by PD1 and CD153 expression that accumulate in spleen with age and adipose tissues in obese model (Shirakawa et al., 2016). This cell population express various senescence gene signature including expression of osteopontin that results in inflammation.

Interestingly, the retrotransposon LINE-1, is expressed at higher levels in dysfunctional T cells. Reduction in the number of LINE1-containing transcripts restores the effect function of T cells. Although LINE-1 is known to be upregulated in senescence cells, leading to a type 1 IFN response, it has been suggested that the level of LINE-1 expression is important for modulating T cell quiescence, effector function and exhaustion (Marasca et al., 2022).

### Senescence in B Cells

A senescence signature including increased SASP also has been demonstrated in B cells from adipose tissue as compared to B cells in the blood (Frasca et al., 2021). Also, in the periphery, memory B cells were shown preferentially to express SASP markers (Frasca et al., 2021). Similarly, in a mouse model of accelerated immune age and in naturally aged, splenic B cells had increased *p16* ^*INK4a*^ and *p21* ^*Cip1*^ expression, but with a limited SASP as compared to other immune subtypes (Yousefzadeh et al., 2021). In mice, there is an expansion of a subset of B cells that accumulate with age referred to as age-associated B cells (ABC) (Cancro, 2020). These cells have been phenotypic defined as B220^lo^CD19^+^ but negative for CD21 and CD23, which are markers of mature B cells. They have also been shown to express CD11c and T-bet. T-bet drives these B cells towards a pro-inflammatory phenotype. ABC produce more IFNγ and TNF-α and drive the differentiation of CD4^+^ T cells to Th17 cells. ABC have been shown to suppress the development of B cells in the bone marrow which was linked to TNF-α secreted by resident ABC (Ratliff et al., 2013). These results suggest that the senescence-like phenotype in B cells and likely other immune cell types depends on their environment and/or function.

### Senescence in Myeloid Cells

Macrophages and neutrophils are phagocytes that remove pathogens from the body. With age, their protective phagocytic functions decline, leading to disruption of tissue homeostasis. For instance, macrophages and neutrophils in the aged bone marrow that produce grancalcin have been shown to display inflammatory and senescence gene expression patterns. Grancalcin released from these senescent cells induces premature bone aging by reducing osteogenesis and increasing adipogenesis. The types of these grancalcin-expression cells include macrophages and neutrophils (Li et al., 2021) Aged macrophages also show M1-like phenotypes with pro-inflammatory gene expression, leading to delayed fracture healing (Clark et al., 2020). Senescence-related molecules might be involved in age-associated changes of macrophages because p16^INK4a^ deficiency drive macrophages to M2 (Cudejko et al., 2011). However, it also should be noted that expression of p16^INK4^ reduced LPS-induced IL-6 expression by inhibiting AP-1 pathway in macrophages but not fibroblasts (Murakami et al., 2012). In addition, SA-β-gal + mast cells that increase with age produce CXCL1, causing aberrant neutrophil trafficking to remote organ such as lungs rather than local injured tissues. These neutrophils cause lung damage (Barkaway et al., 2021).

Non-classical monocytes also accumulate with age. They show senescence features including shorten telomere length, reduced proliferation and SASP factor secretion (Ong et al., 2018). In severe COVID-19 patients, non-classical monocytes as well as inflammatory transitional monocytes are recruited to lungs (Sánchez-Cerrillo et al., 2020). The observation implies that senescent inflammatory monocytes that accumulate with age might explain the mechanism of high mortality in elderly COVID-19 patients.

## Cellular Senescence of the Immune System in COVID-19

The COVID-19 pandemic revealed that age is the most important risk factor contributing to the severity and mortality of the infection. Epidemiological surveys tracking characteristics linked to the severity and mortality of COVID-19 are similar to those associated with aged immune system. For example, PD-1^+^ exhausted T cells are dramatically increased in aging (Lee et al., 2016). In COVID-19 patients, increases in PD-1^+^ memory T cell frequency were observed both in patients with mild and severe symptoms, relative to healthy volunteers, with the more severe cases having a higher percentage (Diao et al., 2020; Mahmoodpoor et al., 2021). An increase of PD-1^+^CD44^+^ memory T cells was observed in mice with Ercc1-deleted immune cells, implying that the senescence status caused by DNA damage accumulation might affect T cell exhaustion (Yousefzadeh et al., 2021). SARS-CoV-2 infection also induces an increase frequency of T cells expressing markers of senescence, CD57 (De Biasi et al., 2020). In addition, cytotoxic T cell activity is compromised in severe COVID-19 patients (Vigón et al., 2021).

Severe SARS-Cov-2 patients also have been shown to suffer from hyper-inflammation driven largely by the increase in cytokines produced by immune cells, which has been term cytokine storm (Hu et al., 2021). An increase of MCP-1, a SASP factor, is associated with mortality in COVID-19 infection and the genetic or pharmacological clearance of senescent cells from aged mice improved the survival against ß-coronavirus infection (Abers et al., 2021; Camell et al., 2021). Also, there appears to be increase in SnCs that is maintained following clearance of the ß-coronavirus, suggesting that SnCs could contribute to long-hauler syndrome (Tsuji et al., 2022).

## Conclusions and Perspectives

It is clear that immune function is impaired with aging, a phenomenon termed as immunosenescence, leading to more severe infection with pathogen and increased mortality. There is also evidence of increase senescence in multiple types of immune cells with age as well as following pathogen exposure. In fact, it is likely that immune senescence as well as the overall senescent cell burden are key pathogenic components following exposure to viral and bacterial microbes including SARS-CoV-2. Several recent studies demonstrated that reducing the senescent cell burden and the inflammatory SASP by treatment with senolytic compounds improves the immune response and reduces mortality (Camell et al., 2021; Lee et al., 2021; Verdoorn et al., 2021; Tsuji et al., 2022). These observations have led to several clinical trials to test senolytic compounds in COVID-19 patients. It is also possible that reducing the senescent cell burden can improve the immune responses to vaccines.

Interestingly, exposure to pathogens can increase the extent of senescence through both direct and indirect mechanisms, especially in the elderly, driving further immune dysfunction, senescence and non-specific inflammation. This increase in inflammation driven by the SASP then contributes to increased mortality and morbidity. Although this is likely also true in younger individuals, the more functional, younger immune system can clear senescent cells, thus maintaining a low senescent cell burden and thus low inflammation. Importantly, these observations suggest that developing approaches to limit senescence in the adaptive and innate immune cells would not only improve the immune response, but also slow aging.
